# Bioextractive Removal of Nitrogen by Oysters in Great Bay Piscataqua River Estuary, New Hampshire, USA

**DOI:** 10.1007/s12237-019-00661-8

**Published:** 2020-01-01

**Authors:** Suzanne B. Bricker, Raymond E. Grizzle, Philip Trowbridge, Julie M. Rose, Joao G. Ferreira, Katharine Wellman, Changbo Zhu, Eve Galimany, Gary H. Wikfors, Camille Saurel, Robin Landeck Miller, James Wands, Robert Rheault, Jacob Steinberg, Annie P. Jacob, Erik D. Davenport, Suzanne Ayvazian, Marnita Chintala, Mark A. Tedesco

**Affiliations:** 1 National Centers for Coastal Ocean Science, Silver Spring, MD 20910, USA; 2 Present address: School of Oceanography, University of Washington, 7616 Latona Avenue NE, Seattle, WA 98115, USA; 3 Department of Biological Sciences, Jackson Estuarine Laboratory, 85 Adams Point Road, Durham, NH 03824, USA; 4 New Hampshire Department of Environmental Services, Durham, NH 03824, USA; 5 Present address: Connecticut Department of Energy and Environmental Protection, 79 Elm St, Hartford, CT 06106, USA; 6 NOAA Fisheries NEFSC Milford Laboratory, 212 Rogers Avenue, Milford, CT 06460, USA; 7 Present address: Institute of Marine Sciences (ICM-CSIC), Pseig. Maritím Barceloneta 37-49, Barcelona 08003, Spain; 8 Department Environmental Sciences & Engineering, Faculty of Sciences and Technology, Universidade Nova de Lisboa, Lisbon, Portugal; 9 Present address: National Institute of Aquatic Resources, Øroddevej 80, Nykøbing Mors 7900, Denmark; 10 Northern Economics, Inc., 1455 NW Leary Way, Suite 400, Seattle, WA 98107, USA; 11 HDR | HydroQual, 1200 MacArthur Boulevard, Mahwah, NJ 07430, USA; 12 East Coast Shellfish Growers Association, 1121 Mooresfield Road, Wakefield, RI 02879, USA; 13 EPA, ORD, National Health and Environmental Effects Research Laboratory, Atlantic Ecology Division, 27 Tarzwell Drive, Narragansett, RI 02882, USA; 14 EPA Long Island Sound Office, Government Center, Suite 9-11, 888 Washington Boulevard, Stamford, CT 06904-2152, USA

**Keywords:** Bioextraction, Ecosystem service, Nutrient removal, Economic valuation, Oyster production model

## Abstract

Eutrophication is a challenge to coastal waters around the globe. In many places, nutrient reductions from land-based sources have not been sufficient to achieve desired water quality improvements. Bivalve shellfish have shown promise as an in-water strategy to complement land-based nutrient management. A local-scale production model was used to estimate oyster (*Crassostrea virginica*) harvest and bioextraction of nitrogen (N) in Great Bay Piscataqua River Estuary (GBP), New Hampshire, USA, because a system-scale ecological model was not available. Farm-scale N removal results (0.072 metric tons acre^−1^ year^−1^) were up-scaled to provide a system-wide removal estimate for current (0.61 metric tons year^−1^), and potential removal (2.35 metric tons year^−1^) at maximum possible expansion of licensed aquaculture areas. Restored reef N removal was included to provide a more complete picture. Nitrogen removal through reef sequestration was ~ 3 times that of aquaculture. Estimated reef-associated denitrification, based on previously reported rates, removed 0.19 metric tons N year^−1^. When all oyster processes (aquaculture and reefs) were included, N removal was 0.33% and 0.54% of incoming N for current and expanded acres, respectively. An avoided cost approach, with wastewater treatment as the alternative management measure, was used to estimate the value of the N removed. The maximum economic value for aquaculture-based removal was $105,000 and $405,000 for current and expanded oyster areas, respectively. Combined aquaculture and reef restoration is suggested to maximize N reduction capacity while limiting use conflicts. Comparison of removal based on per oyster N content suggests much lower removal rates than model results, but model harvest estimates are similar to reported harvest. Though results are specific to GBP, the approach is transferable to estuaries that support bivalve aquaculture but do not have complex system-scale hydrodynamic or ecological models.

## Introduction

Nutrient load reductions have been mandated in the USA (US; Clean Water Act) and in the European Union (EU; Water Framework Directive) to alleviate estuarine and coastal water eutrophication impacts such as excessive algal blooms, hypoxic bottom waters, and loss of seagrasses (Bricker et al. 2007; Zaldivar et al. 2008). Management has primarily targeted land-based sources of nutrients including maximizing efficiency of nutrient removal from wastewater treatment plants (WWTP; e.g., Kessler 2010), implementing best management practices (BMPs; e.g., Evans 2008) to reduce nutrient runoff from agriculture and stormwater, and regulation of US electrical generating units to reduce atmospheric inputs (e.g., Linker et al. 2013; Eshleman et al. 2013). In some cases, these measures have been successful and water quality improvements were observed. In Long Island Sound, a 40% reduction in wastewater nitrogen (N) inputs from 1995–2013 resulted in increased bottom-water dissolved oxygen (DO; Latimer et al. 2014; LISS 2013). A 50% N load reduction to Tampa Bay since 1995 led to the recovery of seagrasses to 1950s levels (Sherwood et al. 2017; Greening et al. 2014). Implementation of improved WWTP processes in Venice Lagoon resulted in regrowth of seagrasses and reductions of macroalgal blooms (Pastres et al. 2004; Sfriso and Marcomini 1996). In Boston Harbor, elevated nutrient loads were reduced by diversion of effluent 15 km offshore for diffusion into bottom waters of Massachusetts Bay leading to an observed 40% decrease in average chlorophyll (CHL) concentrations and increases in mid-summer DO concentrations (Taylor 2006). In Narragansett Bay, successful improvements to WWTP led to a 75% decline in discharge of total suspended solids (TSS) from 1983–1995 and a coincident 50% reduction in CHL concentrations (Borkman and Smayda 2016). A further 50% reduction of WWTP dissolved inorganic nitrogen (DIN) from 2005 to 2012 resulted in a 60% decrease in concentration of total nitrogen (TN) in bay waters, improved water clarity, a 34% reduction in summer hypoxia, and 31% and 45% decreases in apparent production in upper and mid Narragansett Bay, respectively (Oviatt et al. 2017).

In other locations, nutrient source reductions have not resulted in achievement of water quality goals (Kemp et al. 2009). Measures taken to reduce point source loads in Denmark have been successful, with WWTP N loads decreasing 74% from 1989–2002, but expected improvements in bottom-water DO concentrations have not been observed perhaps as a result of hypoxia-related ecosystem changes in susceptibility (Conley et al. 2002, 2007). Modeling efforts in the Gulf of Mexico have also indicated an increasing sensitivity of the system to N loading, which would suggest greater N reductions may be necessary than originally anticipated (Liu et al. 2010). Overall, there is increasing recognition that coastal populations and nutrient discharges will continue to rise but returns on investment diminish with increased stringency in both point and non-point source controls (Stephenson et al. 2010). A variety of nutrient management tools, targeting both point and non-point sources of nutrients, are needed. In most watersheds, no single method of nutrient reduction will be enough to achieve water quality goals.

Management programs are considering innovative ways to treat nutrients in surface or ground waters before they can result in adverse impacts to water quality (Cape Cod Commission 2015). Removal of nutrients directly from the water by cultivation of bivalve shellfish has also shown promise as a complement to land-based measures (Lindahl et al. 2005; Jones et al. 2010; Rose et al. 2014; Petersen et al. 2016; Ferreira and Bricker 2016). Bivalves filter water as they feed and remove N contained in plankton and particulate detrital matter transferring N from the water column to the sediments as feces or pseudofeces, sequestering some N in tissue and shell and excreting the balance as ammonia (NH_4_). Their feeding activities effectively short-circuit accumulation and degradation of organic matter and consequent reduction of bottom-water DO, as well as improving water transparency, allowing for regrowth of seagrasses—except in cases where over population of oysters or low current speeds exist. Nutrients are removed through sequestration of assimilated food into shell and tissue which removes nutrients from uptake by phytoplankton, through oyster-related denitrification in associated sediments, and through burial in sediments (Officer et al. 1982; Humphries et al. 2016).

It is important to note that appropriate siting and farm management are needed to maximize environmental benefits and avoid any potential negative environmental effects associated with bivalve aquaculture. Over-enrichment of sediments underlying aquaculture operations can occur in locations with small or no tidal currents, and/or excessively high densities of shellfish in the farm area (Lindahl et al. 2005). High rates of biodeposition without sufficient dispersal in these areas can lead to DO depletion as organic matter decomposes, and may result in N release from sediments (Higgins et al. 2013). In cases where organic matter settles on oxygenated sediments, NH_4_ released from decomposition may be oxidized to nitrite (NO_2_) and nitrate (NO_3_) during nitrification. Some NH4 may be converted to nitrogen gas (N_2_) through denitrification in the coupled nitrification-denitrification process (Testa et al. 2015; Newell et al. 2005). In other cases, depletion of DO near the sediment–water interface allows sulfide accumulation and can inhibit nitrification allowing regenerated N to remain in the system as NH_4_, potentially supporting algal and microbial production (Testa et al. 2015; Newell 2004).

**Fig. 1:**
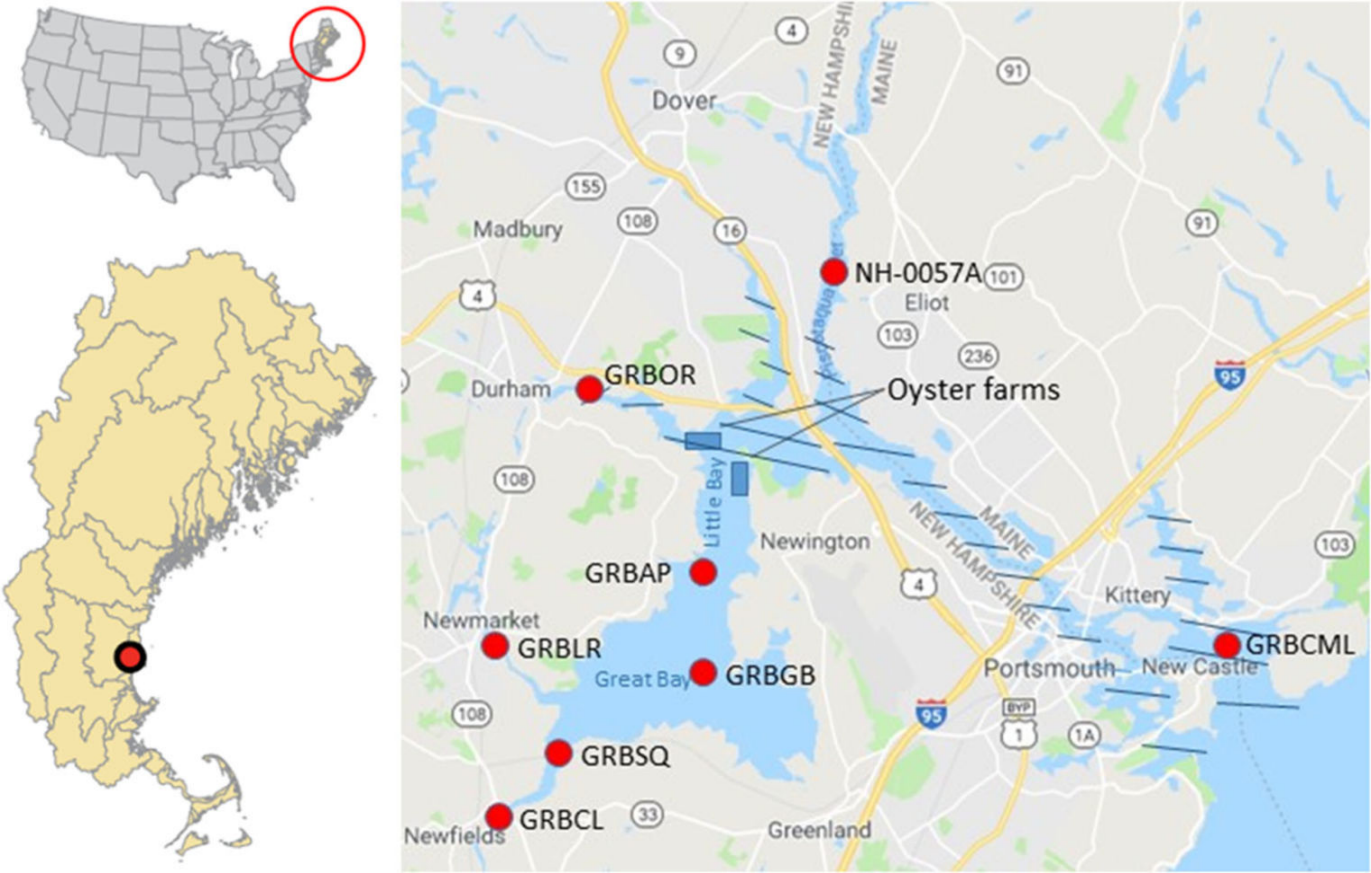
Map of Great Bay Piscataqua River Estuary (right). Sampling stations marked by red dots, note that data from station GRBAP (at Adam’s Point) were used for modeling. The seawater zone (> 25 PPT annual and depth averaged) is marked with blue hatchmarks, the rest is considered mixing zone (0.5–25 PPT annual and depth averaged; Bricker et al. 1997). Simulated oyster farm locations in Little Bay are shown. Inset maps of Great Bay Piscataqua River Estuary location within the northeast region of the US (left)

The focus of this study was the quantification and valuation of the ecosystem service of N removal provided by cultivated Eastern oysters (*Crassostrea virginica*) at present production (at the time of this study, 2014) and the potential increase in services in expanded production to maximum allowable cultivation areas in Great Bay Piscataqua River Estuary (GBP). As there is no 3D hydrodynamic or ecological model available for this waterbody, we used an approach that estimates oyster production (harvest) and N removal at the local scale then used those rates to upscale to licensed aquaculture areas given specific assumptions. This approach was tested in Long Island Sound where we compared results from this local-scale approach to results from a system-wide 3D hydrodynamic ecological model; upscaled farm-scale results compared favorably with ecosystem model results (Bricker et al. 2018). We suggest that this approach will be useful in places like GBP where a complex ecological model is not available. The estuary is shared by New Hampshire (NH) and Maine (ME; Fig. 1), but the section of the estuary that was modeled for oyster farming was in NH. The focus was N because it is typically the limiting nutrient in estuarine waterbodies and has been the focus of coastal nutrient management (Malone et al. 1996; Howarth and Marino 2006). The analysis was extended to include restored reefs to provide a more complete picture of oyster-related N removal ecosystem services. GBP is included in the 65% of US estuaries that have previously been shown to have moderate to high level eutrophication impacts (Bricker et al. 2007; NH DES 2010; PREP 2018).

The objectives of this study were to: 1) determine the mass of N removed through oyster cultivation at current and expanded aquaculture production and by restored reefs; 2) assess how significant the removal is in relation to the total N loading under current and expanded production scenarios; 3) estimate the economic value of the ecosystem service of N removal being provided by oyster aquaculture and restored reefs.

## Study Site, Oyster Cultivation, and Restoration Practices

The GBP is an estuarine system of 54.7 km^2^, located between NH and ME in the northeastern USA (Fig. 1). It is composed of the Piscataqua River, Little Bay, and Great Bay areas, and includes the Great Bay National Estuarine Research Reserve (GBNERR 2011). It has been designated as an estuary of national significance under the US EPA National Estuary Program. Eight major rivers, several small creeks, and their tributaries drain into the estuary. GBP is tidally dominated and the water column is well mixed due to tidal height (mean 2.0–2.6 m) and basin geometry, though there can be localized moderate stratification at times of high freshwater inflow. Mean depth is 4 m and mean salinity is 21 PPT (Bricker et al. 1997). The 2651 km^2^ GBP watershed area includes parts of 57 towns in ME and NH with a population of ~ 288,000 in 2010 (PREP 2013). Noted for its valuable water and cultural resources, business and industry, the GBP Region is very important to state and local economies.

The 2013 and 2018 State of the Estuary reports showed that GBP exhibited many classic symptoms of eutrophication including low DO in tidal rivers, excessive macroalgae growth, and occurrences of nuisance and invasive macroalgal species (PREP 2013; 2018). Of major concern was the loss of eelgrass which had declined by 35% since 1996 (Short 2011). Consistent with the PREP (2013) conclusions, two national assessments and a study of northeast estuaries showed GBP eutrophication status as moderate (Bricker et al. 1999, 2006, 2007). A more recent analysis of eutrophication showed that conditions are moderately high (Bricker et al. 2015). The latest State of the Estuary report (PREP 2018) concluded that low DO events occur in all rivers, macroalgae cover continues to increase, and eelgrass area continues to decrease. In 2009, observed water quality issues led to inclusion of GBP in the EPA 303d list of N impaired waters. Inclusion in the impaired waters list led to a load reduction analysis to determine the level of N loads needed to maintain desired water quality, and development of guidance for meeting water quality goals through specific N reductions (NHDES 2009). Based on simplified models, the analysis determined that a 30–45% reduction of N loads to GBP would be needed to achieve water quality goals (NHDES 2010).

The average annual N load to GBP in 2009–2011 was 1110 metric tons and was 819^1^ metric tons in 2012–2016. The decrease was attributed to reductions of municipal WWTP discharges and low rainfall in recent years (PREP 2013, 2018). Sixty-eight percent of total N inputs were from non-point sources including fertilizer from lawns and farms, septic systems, animal wastes, and atmospheric deposition onto the watershed that are delivered to GBP through rain and snowmelt runoff, river, and groundwater flow. The balance of N was discharged to GBP or to tributary rivers from 18 municipal sewage treatment plants (PREP 2013, 2018). Major N contributors were related to population growth and associated building and development patterns within the watershed. Of great concern was that projected continued increase in non-point N loads would nullify the impact of ongoing WWTP upgrades (PREP 2013, 2018). Restoration of oyster reefs and expansion of oyster aquaculture were also studied as a potential complement to traditional land-based N management measures in GBP (Nash and Elliott 2012; Konisky et al. 2014). The load reduction analysis suggested that a percentage of the non-point source N load could be attenuated through increasing the assimilative capacity of the waterbody, such as N reductions achieved by restoring wetlands and through bioextraction by shellfish. Determining the value of the ecosystem services of nutrient reduction provided by these innovative management techniques would provide justification of their cost to decision-makers and help with development of a successful, cost-effective nutrient management program.

The shellfish aquaculture industry in GBP at the time of this study (2014) was relatively small but continues to grow and has remained focused primarily on Eastern oysters. In 2014, there were 25.5 acres licensed for aquaculture which grant growers permission (but not exclusive rights) to use public lands (land below the mean high tide line is public trust land in NH). No farm is greater than 4.5 acres. Wild sets of oyster larvae are sporadic, thus growers rely on hatchery-reared seed oysters that have been selectively bred for fast growth and disease resistance. Growers are able to plant at relatively high densities (100–200 m^−2^ in mesh bags within bottom cages) as long as algal concentrations and current speeds are both high enough to support good growth. A 3-inch (76 mm) harvest size diploid^2^ oyster can be grown in 3 years (Grizzle et al. 2016). Oyster growers in GBP typically use a rotational scheme where 1/3 of the licensed acres are planted with seed, 1/3 of the acres have 2-year-old oysters, and 3-year-old harvest size oysters are harvested from the remaining 1/3 of the farm acres.

In 2014, growers received $0.55-$0.65 wholesale per oyster, typically from the half shell market. For every oyster sold in 2014, the grower paid $0.015 to the state to support the general funds of the NH Department of Fish and Game (NH F&G). Additionally, growers paid a $100 annual fee for licenses and certifications, as well as a $200 per acre fee for their use of public land (NHDES Shellfish Program, undated). The GBP industry is small and other operational cost information was not available. However, a recent study in Chesapeake Bay (Parker 2019) provides some insight to costs that might be incurred by the GBP oyster farm operations (Table 1 in Supplemental Material). All present operations are subtidal. There is interest in expanding to intertidal areas but future expansion will most likely be in bottom culture due to concerns (described as social carrying capacity, Angel and Freeman 2009) by waterfront landowners and boaters who do not want to see floating gear or risk boats getting entangled in aquaculture gear. No harvest numbers are available for 2014, the year of the study, but the oyster aquaculture industry reported oyster farm landings for 2017 of 330,000 oysters with estimated dockside value of $250,000 (Robert Atwood, NH F&G, Pers. Comm.).

Natural oyster populations in GBP declined from over 25 million adult oysters in 1993 to 1.2 million in 2000 due to disease, sedimentation, and human harvest (Grizzle and Ward 2016). In 2009, work began to restore reefs in order to regain their filtering capacity and other ecosystem services; 26 acres of oyster reef have been restored (PREP 2018). The restoration method used in GBP was “spat seeding” in which larvae from disease resistant and/or fast growing diploid broodstock was set onto cultch material (e.g., aged oyster shells) in large shore-based tanks. The cultch material with attached spat was spread at the bottom and the restored reef was allowed to grow undisturbed; harvest of reefs is prohibited (Grizzle and Ward 2016). Restoration of one acre of reef including permitting, oyster larvae purchase, remote setting nursery raft, reef base construction, and spat seeding cost $54,800 (Grizzle et al. 2006; Table 2 in Supplemental Material) compared with $25,000 acre^−1^ restored in Maryland Chesapeake Bay for the same method (MD Sustainable Fisheries Goal Implementation Team 2015).

## Materials and Methods

Field data from a local monitoring program (2005–2010) and knowledge of local industry practices were used as inputs to run the Farm Aquaculture Resource Management (FARM) model (Ferreira et al. 2007). To provide a more comprehensive picture of the potential impact of all GBP oysters on N removal, restored oyster reefs were included in the calculation of removal through sequestration of N into tissue and shell and reef-associated denitrification. An avoided, or replacement, cost economic analysis was used to estimate the value of the ecosystem service of N removal provided by both cultivated oysters and restored oyster reefs. A similar approach was used previously in Potomac River (Bricker et al. 2014) and in Long Island Sound (Bricker et al. 2018). Additional methodological and analytical details can be found in Bricker et al. (2015).

### The Farm Aquaculture Resource Management Model

The Farm Aquaculture Resource Management (FARM) model combines physical and biogeochemical, bivalve growth, and eutrophication screening models for determining shellfish harvest and for eutrophication assessment (Ferreira et al. 2007). The model evaluates the potential for oyster aquaculture to reduce eutrophic symptoms without the cost and time required for implementation of a farm (Figs. 1, 2, 3, and 4 in Supplemental Material). Water quality inputs to the model include monthly measurements of temperature, salinity, TSS, particulate organic matter (POM), CHL, and DO. The model can be applied to suspended culture (rafts or longlines), as well as to bottom culture. Here we simulated bottom culture because the industry currently uses bottom cages as their primary cultivation practice and industry expansion will most likely be bottom licenses due to social carrying capacity concerns noted above.

The FARM model is well described and has been tested in the USA, EU, China, and elsewhere (Ferreira et al. 2007, 2008, 2009, 2011, 2012; Nunes et al. 2011; Bricker et al. 2014, 2015, 2016, 2018; Saurel et al. 2014). The FARM model calibrated for Long Island Sound was used for this analysis. An individual model for the Eastern oyster (*Crassostrea virginica*) was developed for Long Island Sound (AquaShell, Bricker et al. 2018) and was incorporated into the FARM model to simulate population growth (Fig. 3 in Supplemental Material). It is assumed that there is no interaction (i.e., potential food depletion, e.g., Filgueira et al. 2015) with adjacent farms. The FARM model is useful for analysis of farm-scale aspects of nutrient drawdown and estimation of credits for nutrient credit trading purposes. The output of greatest interest is the net mass of N removed by oyster uptake of phytoplankton and detritus (i.e., food for the oysters) and assimilation into tissue and shell, minus N returned to the environment through pseudofeces and feces, mortality, and excretion (Figs. 1, 2, 3, and 4 in Supplemental Material). Other model outputs include the harvestable biomass of oysters, people equivalents (PEQ) of N which assume an annual per person N load of 3.3 kg, and changes in CHL, DO, and NH_4_ concentrations attributable to the oyster farm operation (Ferreira et al. 2007).

Typical bottom culture practices for Eastern oysters employed by GBP growers described above were used for the simulation. Model simulations were done at two sites using water quality data from station GRBAP (Fig. 1) and current speeds measured at the two locations (maximum spring currents of 0.60 and 0.29 m sec^−1^ and maximum neap currents of 0.47 and 0.19 m sec^−1^). Five model simulations were made using different mortalities (55%, 65%, 75%, 85%, 95%) to represent the range of potential N removal given mortalities observed at GBP farms (55%−95%).

To provide a comprehensive picture of all oyster-related removal, we extended the analysis to include N removal by restored oyster reefs, which we assume have the same N removal rate via sequestration into tissue and shell as bottom aquaculture oysters (Kellogg et al. 2014; Cornwell et al., in prep). While not harvested, oysters in the reef are still sequestering N into tissue and shell which removes it from the water column and from active uptake by algae. In a successfully restored reef, while individual oysters may die, the total live oyster biomass remains greater than what existed prior to restoration efforts, and the overall N reduction is maintained. A recent legal opinion from EPA Region 3 Regional Counsel, in cooperation with EPA General Counsel, indicated support for crediting N reductions associated with reef restoration under the Clean Water Act (Reichert-Nguyen 2018; Cornwell et al., in prep). In 2014 there were 26 acres of restored reef in GBP (PREP 2018).

The FARM modeled N removal estimates do not include denitrification, which was not measured in this study. Denitrification rates are highly variable and site specific, ranging from 0.98 to 295 kg acre^−1^ year^−1^ (Humphries et al. 2016; Lunstrum 2015) and rates for restored reefs are better constrained than for aquaculture (Cornwell et al., in prep; Testa et al. 2015; Higgins et al. 2013; Kellogg et al. 2013). In July 2019, denitrification associated with restored oyster reefs was conditionally approved as a BMP by the Chesapeake Bay Program (WQGIT 2019); thus, we focus on denitrification only from reefs. A previously reported value measured in GBP was used with the acres of restored reef to provide an estimate of denitrification N removal. Previously measured rates from a river mouth site (7.44 kg acre^−1^ year^−1^) were used because most restored reef locations are located within tidal rivers (Hoellein et al. 2015; Group et al. 2018). The river mouth denitrification rates were ~ 2 times higher than at a site in the middle of GBP, and both were about an order of magnitude less than rates measured in a Rhode Island waterbody (Humphries et al. 2016).

### Monitoring Data

Data used for FARM model inputs were sampled by the GBNERR System-Wide Monitoring Program and the University of New Hampshire (UNH) Tidal Water Quality Monitoring Program. Monthly mean data for 2005–2010 from station GRBAP were used for model inputs, the years of data that were fully analyzed for quality control/quality assurance at the time of the study. This station was selected from the 8 GBP sampling stations that had adequate data because it is the closest to the oyster farms that were used for the simulations and is the representative of water quality in the bay (Fig. 1, Table 1). Also, this station is closest to current and potential future oyster farming locations. Statistical Jonckheere-Terpstra tests (JT; Zar 1999) were performed to detect trends at each station that could potentially bias results. All p values were greater than the standard a-level of 0.05 indicating that there was no trend for any parameter, at any station during the 2005–2010 data series.

Water quality concentrations at station GRBAP vary annually, but most concentrations fall within the range of values measured at the other stations (Table 1). Analysis shows that the 10th percentile of annual DO grab sample concentrations, are all above 5 mg L^−1^, the threshold considered protective of all organisms (PREP 2018). Analysis of datasonde records for DO, taken every 15 min, shows that station GRBLR had 10th percentile DO values of 4.0 mg L^−1^ during 2005–2010. These results are consistent with the 2018 State of the Estuary report showing that concentrations were typically not of concern, but that low DO events (< 5 mg L^−1^) did occur for short periods on a weekly basis in late summer in tidal portions of tributary rivers (PREP 2018). Annual mean CHL concentrations were below 20 g L^−1^ at all GBP stations and were below 5 μg L^−1^ at 5 of 8 stations (Table 1). This is consistent with PREP (2018) results which noted episodic concentrations > 20 μg L^−1^ at some stations. The 90th percentile of annual CHL data showed that most stations had concentrations in the moderate or fair category (5–20 μg L^−1^; Bricker et al. 2003), none were in the high category. Data for station GRBAP falls within the range of values observed at other GBP stations during 2005–2010, thus the data were used as representative of GBP conditions with confidence.

### Economic Framework

Converting the benefits of ecosystem services to a common comparable unit (dollars) represents a major challenge to economists (Peterson and Lipcius 2003). To estimate the value of removed N in GBP, we applied an avoided, or replacement, cost approach used recently in Long Island Sound (Bricker et al. 2018). This approach assumes that there is equivalency of N removal services, that the avoided cost good is the least cost for N removal, and that there is willingness to pay because of the inclusion of GBP on the 303d list for N impairment and requirement of N load reductions to meet water quality goals (Freeman et al. 2014). This approach assumes that if oysters are no longer harvested, the N removal services they provide would need to be replaced. While WWTP upgrades and agricultural and urban BMPs are the likely candidates to replace the service that oysters provide, only GBP-specific cost data for WWTP were available. Thus, WWTP costs were the focus of the analysis. It is important to note that using only WWTP costs will necessarily underestimate the value of oyster-related N removal. While costs of both point and non-point source N controls vary and are site specific, the costs of non-point source controls are typically much greater than point source reduction strategies as shown by Rose et al. (Rose et al. 2015; Table 3 in Supplemental Material).

Cost information based on increasing the effectiveness of GBP WWTPs were derived from Kessler (2010), who conducted an assessment to determine cost-effectiveness of WWTP upgrades compared with alternative N removal options (e.g., agricultural and urban BMPs) using the approach of Evans (2008; Tables 5, 6 in Supplemental Material). Capital plus operation and maintenance (O&M) costs for removing varying amounts of N at 18 municipal WWTP currently discharging into GBP were evaluated. The O&M costs were based primarily on the 2009 operating budgets from WWTP operators. Additionally, existing operating budgets included estimated costs associated with more advanced treatment levels for N removal (Chesapeake Bay Program 2002). Three N effluent limits were considered: 8, 5, and 3 mg L^−1^. The cost of N removed due to WWTP upgrades was calculated as the annualized cost divided by the kilograms of N removed from the wastewater stream by the WWTP. Amortized capital costs plus annual O&M costs were combined to estimate the total annual costs for each treatment level. A range of interest rates from 2 to 5 percent were selected to bracket potential rates for a 20-year bond. The Engineering News Record Construction Cost index (ENRCC) was used to adjust for inflation to 2013 dollars. Overall, Kessler (2010) reports that if all the WWTPs were given N effluent limits of 8 mg L^−1^ at design flow, a total of 106 metric tons of N per year would be removed, with an average per unit cost of $172 kg^−1^ N^−1^ removed (Table 2). For the 5-mg N L^−1^ limit, 196 metric tons of N removed per year would have an average per unit cost of $150 kg^−1^ N^−1^. The 3-mg N L^−1^ limit would result in removal of 255 metric tons of N with an average cost of $154 kg^−1^ N^−1^ removed.

## Results

### Local-Scale FARM Model Results

The FARM model estimated production (harvest) and N removal results showed no differences between the two representative farm sites; these results represent both sites. The range of oyster filtration-related N removal was estimated to be 0.037–0.101 (mean 0.072) metric tons N acre^−1^ year^−1^, representing a population equivalent of 11–31 (mean 22) PEQ acre^−1^ year^−1^ (Table 3). Results showed that CHL, DO, and NH_4_ concentrations did not change. Harvestable oyster biomass was estimated to be 0.57–5.27 (mean 2.93) metric tons of oysters acre^−1^ year^−1^.

Farm-scale results were scaled up to evaluate current and potential system scale N removal using; 1) current acres of oyster licensed area (25.5 acres in 2014–8.5 acres used per oyster year class) and 2) estimates of maximum expanded cultivation area (98 acres-33 acres used per oyster year class). The analysis for determination of maximum possible expanded acres of oyster growing areas in GBP (Nash and Elliott 2012) used a GIS-based approach that excludes unsuitable area in the manner of Silva et al. (2011). In upscaling the local-scale results, we assumed that 1) oyster growth and N removal rates are the same for all suitable bottom areas in current and expanded cultivation scenarios, 2) there is no food depletion among adjacent farms, though no formal carrying capacity analysis was performed, 3) only one-third of each farm is harvestable at any time, given typical aquaculture practices in this region (Grizzle et al. 2016). Given the aforementioned assumptions, potential N removal on currently licensed areas removes 0.31–0.86 (mean 0.61) metric tons N year^−1^ and removal could be as high as 1.20–3.30 (mean 2.35) metric tons at maximum expanded licensed acres. These estimates correspond to land-based N removal for 94–260 (mean 187) people equivalents (PEQs) and up to 367–1001 (mean 713) PEQs for current and potential production, respectively. The removal is equivalent to 0.075% and 0.29% of incoming N loads (819 metric tons year^−1^) under current and expanded production, respectively.

Restored reefs potentially remove an estimated 0.96–2.63 (mean 1.87) metric tons year^−1^ through assimilation into tissue and shell. This removal nearly quadruples the current N removal to a mean of 2.48 metric tons year^−1^ and increases to a maximum of mean of 4.23 metric tons year^−1^ under the expansion scenario. This represents an increase to an equivalent of 0.30% and 0.52% of incoming N loads. The additional N removal from reef-related denitrification is 0.193 metric tons year^−1^ (Table 3).

The total removal by sequestration into tissue and shell of aquaculture and reefs plus denitrification by reefs represents a maximum removal of 2.68 metric tons N year^−1^ for current areas (25.5) of cultivation, the equivalent of 0.33% of incoming annual N loads. If GBP reaches expansion to 98 acres of licensed cultivation areas, the total N removal increases to a maximum of 4.42 metric tons, the equivalent of 0.54% of incoming N loads.

### Ecosystem Service Valuation

Annualized cost estimates for removal of 1 kg of N via WWTPs at three levels of treatment (Table 2) were applied to the estimated current and potential N removal from oyster farm operations. The different levels of wastewater treatment represent a range of costs that depend on the status of the specific WWTP. For example, the 8-mg L^−1^ level would not apply to a treatment plant that is already meeting a 5-mg L^−1^ effluent limit. Since this analysis evaluated WWTPs in aggregate as a basic measure of avoided cost, the cost of removing N from the wastewater is best represented as a range based on $150 (minimum) and $172 (maximum) kg^−1^ year^−1^.

The annual cost to replace the bioextractive removal of N is estimated to range from $92,000 to $105,000 year^−1^ under the current licensed area scenario depending on the level of WWTP treatment. Avoided cost estimates under the expanded production scenario range from $353,000 to $405,000 year^−1^. The calculated per acre per year value for each scenario shows the same range, $3600 to $4128. The maximum ecosystem service value of N removal increases to an estimated $427,000 and $727,000 for current and expanded acres, respectively, when restored reef N removal via sequestration is included. The maximum potential value, when all oyster processes (i.e., assimilation by aquaculture and reef oysters plus denitrification in reefs) are included, are $461,000 and $760,000 for current and expanded areas, respectively (Table 3).

## Discussion

Since the first evaluation of the use of mussels for nutrient reductions in Sweden by Lindahl et al. (2005), the concept of shellfish cultivation for water quality improvement has gained support. In many places, bivalve shellfish aquaculture (e.g., oysters, clams, mussels) and/or oyster reef restoration has shown promise in reducing eutrophication impacts (e.g., EU, Ferreira et al. 2007, 2009, 2011; US, zu Ermgassen et al. 2013; Chesapeake Bay, Newell 2004; Cerco and Noel 2007; Kellogg et al. 2014; Carmichael et al. 2012; North Carolina, Grabowski and Peterson 2007; Hicks et al. 2004; Texas, Pollack et al. 2013; Massachusetts, Reitsma et al. 2017; Long Island Sound, Bricker et al. 2018; Rhode Island, Humphries et al. 2016). Studies have shown that areal N removal efficiencies by shellfish aquaculture (45–615 kg acre^−1^ year^−1^) are comparable with removal by existing agricultural (0.018–5.25 kg acre^−1^ year^−1^) and stormwater (0–450 kg acre^−1^ year^−1^) best management practices (BMPs), and that the cost per unit removed also compares favorably with approved BMPs (Stephenson et al. 2010; Rose et al. 2015; Tables 3, 4a, and 4b in Supplemental Material).

In Denmark, mussel installations are used specifically for nutrient removal and eutrophication mitigation (Petersen et al. 2014, 2016). In the USA, the Chesapeake Bay Program recently approved the use of harvested oyster tissue (Cornwell et al. 2016) and restored reef denitrification (WQGIT 2019) as nutrient reduction BMPs, and some jurisdictions have already begun to use shellfish to address the legally mandated nutrient reduction requirements. The Mashpee, MA, nutrient management plan includes cultivation and harvest of 500,000 oysters annually that are expected to remove an equivalent of 50% of the required reduction of 5.0 metric tons N in the Mashpee River and Shoestring Bay (Town of Mashpee Sewer Commission 2015; Reitsma et al. 2017). Bioextraction appears to be a promising management strategy in nutrient-impacted waterbodies of all sizes—the Mashpee River complex is < 5 km^2^, the Long Island Sound is 3,300 km^2^, and the Chesapeake Bay region is > 11,000 km^2^ (Bricker et al. 2018). None of the existing or planned nutrient management programs are relying on shellfish as their only nutrient reduction strategy, but all recognize its value as one of the tools available to achieve nutrient reduction goals. Oysters may be a useful additional management tool in GBP given the potential for non-point N discharges to counterbalance WWTP nutrient reduction improvements in this waterbody (Kessler 2010).

### Nitrogen Removal by Oyster Aquaculture

The FARM-estimated N removal rate (mean 0.072 metric tons acre^−1^ year^−1^) is within the range of rates estimated for oyster aquaculture in other waterbodies using other cultivation methods and other oyster species (0.051–0.35 metric tons N acre^−1^ year^−1^; Tables 3, 4). The model predictions of no changes in CHL, DO, and NH_4_ suggest no negative impacts on water quality from the aquaculture operation. This also indicates that there may be margin to increase either oyster seeding density, or the area under cultivation at the same seeding density within GBP. It is possible, however, that higher seeding densities and associated high levels of respiration may cause depletion of DO or reduced individual oyster growth rate if over-populated particularly in areas where the levels of DO are already low. Increased aquaculture-related biodeposition may increase recycling rates locally offsetting N removal (e.g., Newell et al. 2005; Carlsson et al. 2009). Thus, potential expansion of the industry should be carefully planned.

Results of upscaling to licensed aquaculture acres (25.5 acres in 2014) showed that oyster cultivation removed an estimated mean of 0.61 metric tons N year^−1^ or 0.075% of the total input to GBP. This is equivalent to wastewater treatment nutrient removal for 187 PEQs (Table 3). Upscaling to the maximum acres of potential and existing farms (98 acres), assuming that all areas would have the same production and bioextraction capabilities, would lead to an annual oyster-related mean N removal of 2.35 metric tons N year^−1^. This is equivalent to an ecosystem service of nutrient treatment for 713 PEQs (assumes 3.3 kg N person^−1^ year^−1^) and represents a removal of 0.29% of the total N load. Under the currently used seeding density, it would take about 11,000 acres (46 km^2^) in active oyster cultivation to remove the total N input at the estimated removal rate. This is an area equivalent to 80% of the bottom area of GBP. This much area would never be approved for aquaculture given that maximum expansion to the estimated 0.7% area of GBP (98 acres) was determined based on the presence of eelgrass, legal constraints imposed by GBNERR boundaries, mooring fields, and required buffers between adjacent farms (Nash and Elliott 2012). But restoration of reefs could be used in combination with additional aquaculture licenses to provide maximum benefit of oyster-related N reductions while reducing conflicts associated with aquaculture. The current 26 acres of restored reefs are estimated to more than quadruple the calculated N removal for current (2014) aquaculture licensed acres (Table 3).

### Comparison of FARM Results to Independent Estimates

An independent estimate of the amount of harvestable size oysters and associated removal of N in GBP was made based on FARM model harvest estimates and measured N content of local oysters (Grizzle et al. 2016). Typical oyster harvest from a well-managed GBP lease is estimated to be 400–500 100-count bags of oysters acre^−1^ year^−1^ where each bag weighs about 6.8 kg (R. Grizzle Pers. Comm.). This gives an average per oyster weight of 68 g and represents a total harvest from acres being cultivated at the time of the study of 2.7–3.4 metric tons of oysters acre^−1^ year^−1^. This is close to the mean harvest results from the FARM simulation of 2.93 metric tons acre^−1^ year^−1^. Additionally, the AquaShell model (Bricker et al. 2015) simulation for an individual oyster estimates fresh weight of 70 g for an 84 mm oyster, while Grizzle et al. (2016) report the typical measured weight of an 86-mm GBP oyster as 70 g—a favorable comparison of measured and simulated oyster sizes. Thus, the FARM model appears to provide realistic results for growth of bivalves as it has done for other waterbodies (e.g., Long Island Sound, Bricker et al. 2018; Loch Creran, Ferreira et al. 2009).

In the same study, Grizzle et al. (2016) report a range of 0.14–0.32 g of N measured in harvested GBP oysters, which provides the opportunity to compare an independently derived N removal rate to FARM model N removal results. There is some variability among measured mean oyster shell heights in the Grizzle et al. (2016) study which ranged from 83 to 87 mm, while shell and soft tissue dry weight varied by > 50%. Although the percent N in shell also varied by 50%, soft tissue N only ranged from 7.3 to 8.5%. The range of mean total N (0.14–0.32 g) for a regular size oyster appears to be related to variations in weight and N content in shell. Applying Grizzle et al.’s (2016) values for weight of a harvest size oyster to the FARM model harvestable oyster biomass estimates gives a model estimated harvest of 356,000 oysters, close to the 330,000 reported harvest in 2017 (R. Atwood, NH Fish and Game, Pers. Comm.). The estimated N removed by the observed 2017 harvest, based on Grizzle et al. ‘s(2016) measured per oyster N content, is 0.046–0.106 metric tons year^−1^. That estimate is lower than the FARM model estimates of 0.31–0.86 metric tons N year^−1^ removed by current farm acres.

The FARM model estimates N removal via a mass balance approach where N removed is equal to N intake via filtration of particulates and assimilation into tissue and shell minus N in excretion, feces, pseudofeces, and mortality (Ferreira et al. 2007; Figs. 2, 3, and 4 in Supplemental Material). One reason for the greater removal estimated by FARM is that the model estimates removal based on filtration of all oysters planted not just harvested oysters (assuming that not all oysters planted at one time will grow to harvest size by the same date). However, it is unlikely that this would account for such a large difference. Another possibility is uncertainty surrounding the exact areas and seeding densities used by this young and growing industry. It is still small enough that growers were reluctant to provide proprietary information that might be associated with their business. Further, the FARM model N estimates are dependent on calculation of metabolic losses of nitrogen under different conditions, and on conversion factors used internally for energy to nitrogen and energy to oxygen ratios. These process rates and conversion factors should be confirmed experimentally. Finally, within the population dynamics model when oysters reach the highest weight class, harvestable biomass does not increase but the metabolic activities continue which may lead to overestimation of N removal. This type of artifact is common to physiology-based population dynamic models^3^. If FARM is to be used for policy, i.e., estimation of nutrient credits, it will be important to investigate the reason for the differences.

However, there is good concurrence of the measured and modeled shellfish sizes and weights, and harvest is also successfully estimated, suggesting that the FARM model is simulating oyster growth and production (harvest) in GBP reasonably well. Thus, the model can be used for planning purposes. This might include testing scenarios of different aquaculture seeding densities or different areas of cultivation to explore the potential differences in harvest and impact to water quality that might be expected with expansion via higher seeding density or greater area of farm. The FARM-estimated harvest numbers can also be used with the measured oyster N content to estimate N credits under these different scenarios until FARM-estimated N removal calculations have been confirmed.

### Ecosystem Service Value of Nitrogen Removal in Great Bay Piscataqua River Estuary

Using the N removal estimates with avoided cost estimates based on WWTP (Table 2), the value of FARM-based N removal at the 2014 cultivation level is an estimated range of $92,000 to $105,000 per year. If GBP reaches its potential of 98 acres of licensed area, estimated N removal values would increase to $353,000 to $405,000 year^−1^, depending on the level of wastewater treatment used for the analysis. Here we note that the value of N removal may be lower if removal based on a harvested, per oyster N content is used—$7,500–$19,600 for 2014 acres. The values of N removal based on either method of estimation represent costs avoided in terms of additional nutrient abatement options, specifically the three levels of wastewater treatment, and are a proxy for the economic value of N removed through bioextraction. These are low estimates for N removal costs because non-point source removal technologies are much more expensive as noted above (Table 3 in Supplemental Material). These values could be considered to be potential payment to oyster growers in a nutrient credit trading program for the ecosystem services provided by the oyster aquaculture operations (Ferreria and Bricker 2016). While the value of the 2014 harvest is not known, the reported 2017 industry value of $250,000 would increase by more than one-third if growers were paid even the lowest estimate of the value for current nutrient removal services.

### Oyster Aquaculture Bioextraction and Nutrient Management in Great Bay Piscataqua River Estuary and Elsewhere

Nutrient-related water quality degradation continues to be a serious issue in GBP as it is in many global estuaries. An increasing body of work is revealing bioextractive N removal as a promising complement to land-based nutrient management measures. The results of this study show that current and potential oyster aquaculture-related bioextractive N removal in GBP is small compared with the total inputs (0.075–0.29% for 2014 licensed acres and expanded acres, respectively). However, the per acre removal is within the range of efficiencies of approved best management practices for agriculture and stormwater (e.g., early cover crops, crop to forest conversion, wet and dry pond, and gravel wetland stormwater measures; Stephenson et al. 2010; Rose et al. 2015; Tables 4a, 4b in Supplemental Material). Comparison of costs of several categories of N removal strategies shows that aquaculture may be more cost-effective than some other abatement alternatives (Table 3 in Supplemental Material; Stephenson et al. 2010, Rose et al. 2015). These removal rates are based on cultivation of less than 0.5% of the bottom of GBP. Some northeastern states (e.g., Rhode Island, Byron et al. 2011; Delaware, Schwartzkopf et al. 2013) have put upper limits on potential aquaculture areas at 5% of a waterbody area. Though it is unlikely that leases in GBP will be approved beyond the expansion estimated here (98 acres = 0.7% of GBP area) due to the presence of seagrasses, mooring fields, and other legal constraints, an increase to 5% of GBP bottom area would remove 6% of the total N input at current seeding densities. Alternatively, restored reefs could be used in combination with aquaculture to maximize N removal while limiting use conflicts. It may also be possible to increase seeding densities in smaller areas to increase N removal, since current densities do not appear to lead to any decline in water quality. Thus, there could be a greater per acre removal even at current areas of cultivation.

A combination of aquaculture at maximum possible expansion and restored reefs could potentially remove 0.54% of incoming N loads to GBP, which would become a greater percentage as N loads continue to decline with continued management of land-based sources. However, the possibility of aquaculture area expansion is limited and our scenario may overestimate the area of potential expansion if watermen, landowners, and boater protests are able to further limit additional licenses. Additionally, it is important to note that the potential N removal is subject to other unpredictable forces and risks such as hurricanes, diseases, and poaching and/or vandalism which can limit production or destroy a farm or reef completely. Thus, though oysters can remove N and are in some cases already being integrated into nutrient management programs, risks should be acknowledged. An example of integration of risk into a nutrient reduction program that already includes harvest of shellfish is exemplified by the Town of Mashpee, MA, whose management plan assumes that bivalves will self-propagate but includes funds for purchase of seed in the event this does not happen (Town of Mashpee Sewer Commission 2015).

Important to remember regarding the valuation of N removal is that point sources account for only one-third of the total N load to GBP. Since the costs to control non-point sources are typically much higher than the costs to remove N from wastewater, the avoided cost value would likely be much higher. Cost estimates for non-point source N management strategies in GBP are not available but comparison of costs in Long Island Sound, while not a perfect match with respect to land use and population, provides insight. In the CT River Basin, the costs to upgrade WWTP efficiency ranged from $32—$98 kg^−1^ N^−1^ removed (Bricker et al. 2018). Agricultural BMP costs for installation of riparian buffers and cover crops are about $13 kg^−1^ N^−1^ removed. The costs to install urban stormwater BMPs, including construction costs and costs for land acquisition for wet ponds and submerged gravel wetlands (the two most cost-effective BMPs) were $350 kg^−1^ N^−1^ removed. Urban BMPs were 3.5 to > 10 times the cost of WWTP upgrades and were also much greater than costs for agricultural BMPs. But agricultural BMPs may not be appropriate where N reductions are most needed within the CT River Basin. Notably, costs for land acquisition in the CT River Basin are likely much greater than in the GBP watershed thus costs for urban BMPs would likely be lower. These comparisons highlight the possible difference in costs and provide an idea of the potential underestimate of the value of oyster-related N removal based only on WWTP costs.

While shellfish growers are not currently being compensated for the nutrient removal service their shellfish provide, some jurisdictions are already using shellfish for nutrient management (e.g., oysters in Mashpee, MA, Town of Mashpee Sewer Commission 2015; mussels in Skive Fjord, Petersen et al. 2016; Nielsen et al. 2016). And there is movement toward potential payment to growers as evidenced by approval in the State of Virginia of inclusion of shellfish aquaculture in their nutrient trading program (§10.1–603.15:2; VNEAI 2017) and the application of the Oyster Company of Virginia to be a credit aggregator (VA DEQ 2018). Economic valuation highlights the value of the N removal service being provided by oysters and the potential level of compensation that could be distributed to growers if they were to be compensated in a nutrient credit trading program. It also enhances public awareness of the importance of shellfish for water quality improvement in addition to provision of seafood. This study provides support for the bioextraction concept (aquaculture and restored reefs) as a promising additional tool for nutrient management, for use in combination with existing nutrient management measures to help achieve water quality goals. While specific to GBP, this approach can be transferred and used in other estuaries that need nutrient management and support bivalve shellfish populations. Importantly, this approach provides a framework for estimating the potential value of oyster bioextractive removal of N in systems where no ecosystem scale hydrodynamic or ecological model exists.

## Supplementary Material

Supplement1

## Figures and Tables

**Table 1:** Annual means of water quality parameters from monthly measures at 8 stations in Great Bay Piscataqua River Estuary from 2005–2010 (see Fig. 1 for locations). All values for Station GRBAP are within the range of values measured at other stations. Assessment of Estuarine Trophic Status (ASSETS) model CHL assessment criteria (Bricker et al. 2003) are included. The 90th percentile CHL concentrations (in parentheses) represent the highest concentrations observed over an annual cycle where CHL <5 μg L^−1^ is considered good or low, 5–20 μg L^−1^ is considered moderate or fair, and > 20 μg L^−1^ is considered poor or high (PREP 2018). These are the parameters used as inputs to the FARM model

Station	Temp mean (°C)	Temp std dev (°C)	Salinity mean (PPT)	Salinity std dev (PPT)	DO mean (mg L^−1^)	DO std dev (mg L^−1^)	TSS mean (mg L^−1^)	TSS std dev (mg L^−1^)	POM mean (mg L^−1^)	POM std dev (mg L^−1^)	CHL mean (μg L^−1^) 90th percentile) (μg L^−1^)	CHL std dev (μg L^−1^)
GRBAP	11.7	7.32	21.7	6.36	9.57	2.44	19.0	22.1	2.13	1.33	4.15 (7.67)	2.98
GRBCL	14.9	7.73	10.4	8.54	8.62	2.84	30.6	19.6	4.60	2.71	8.51 (14.0)	10.99
GRBCML	11.8	4.17	29.1	2.97	8.32	1.44	15.2	8.70	1.37	0.67	1.71 (2.68)	1.07
GRBGB	15.4	6.44	21.5	7.33	8.69	1.65	18.8	9.80	2.29	1.35	4.86 (9.05)	3.54
GRBLR	15.0	7.50	5.35	8.57	9.77	3.04	5.43	5.52	1.91	1.39	4.85 (7.30)	13.86
GRBOR	16.2	6.58	17.2	8.07	7.52	2.20	19.7	17.3	2.89	2.05	6.02 (9.94)	7.42
NH-0057A	16.3	7.12	11.5	8.36	7.85	2.38	46.1	48.6	5.42	3.74	6.85 (6.10)	6.06
GRBSQ	16.2	6.28	14.1	8.21	8.66	1.82	11.2	6.62	2.22	1.20	3.34 (13.2)	6.80

**Table 2:** Incremental costs and reductions from point source controls at three levels of effluent nutrient concentrations of WWTP upgrades at various levels of nitrogen removal (as 2013 US dollars)

Level	Capital cost ($ millions)	O&M ($ millions)	Annualized cost ($ millions)	Nitrogen removed (kg N year^−1^)	Average cost ($ kg^−1^ year^−1^)
8 mg N L^−1^	215	23.6	16.3–20.2	106,182	172
5 mg N L^−1^	291	28.9	26.1–31.7	195,591	150
3 mg N L^−1^	386	32.4	35.4–42.8	255,273	154

**Table 3:** Range and mean of estimated nitrogen removal by aquaculture and restored reef oysters through sequestration into tissue and shell (aquaculture and restored reefs) based on Famr Aquaculture Resource Management (FARM ) model simulations (this study ), and by denitrification (reefs only ) based on previously measured rates (Grizzle et al. 2006). Also shown is the percentage of incoming N load and the people equivalents (PEQs) represented by oyster-related N removal, and the economic value based on an avoided cost valuation approach (see text). These results are for current (25.5 acres), and maximum possible expanded (98 acres), aquaculture areas and existing restored reefs (26 acres). Note: we assumed the same rate of removal by sequestration into tissue and shell by restored reefs as estimated for bottom grown aquaculture oysters

FARM model estimated farm-scale N removal by Great Bay Piscataqua Estuary oyster farm				0.037–0.101 (mean 0.072) metric tons N removed acre^−1^ year^−1^		
Denitrification removal by restored reefs (based on denitrification measurement by Grizzle et al. 2006)				0.0074 Metric tons N removed acre^−1^ year^−1^		
Measure	Sequestration into tissue and shell			Denitrification	Total (aquaculture and reef sequestration and reef denitrification)	
	Aquaculture		Restored reefs	Restored reefs	Current acres	Maximum acres
	Current acres	Maximum acres				
N removal (metric ton year^−1^)	0.31–0.86 (mean 0.61)	1.2–3.30 (mean 2.35)	0.96–2.63 (mean 1.87)	0.193	1.46–3.68 (mean 2.68)	2.35–6.12 (mean 4.42)
% of incoming load*	0.075	0.29	0.23	0.024	0.33	0.54
People equivalents (PEQ)	94–260 (mean 187)	367–1001 (mean 713)	292–796 (mean 567)	59	445–1115 (mean 833 )	718–1856 (mean 1339)
Economic value range* (10^3^ dollars*)	$92—$105	$353–$405	$281—$322	$29–$33	$402—$461	$663-$760

* based on mean N removal

+ 2013 dollars

**Table 4:** Comparison of Great Bay Piscataqua River Estuary FARM model N removal rates to results in other estuaries

Waterbody	Oyster species	N removal (Kg N^−1^ acre yr^−1^)	Seed density oyster (no. m^−2^)	Ploidy, culture type	Reference
Great Bay Piscataqua River Estuary, NH	*Crassostrea virginica* (Eastern oyster)	72	100	Diploid, bottom or bags	This study
Long Island Sound, CT		105	62	Diploid, bottom or bags	Bricker et al. 2018
Potomac River, MD		230	100	Diploid, spat-on-shell bottom	Bricker et al. 2014
Chester River, MD		81	33	Triploid, cage	Parker and Bricker
Chesapeake Mainstem, MD		87	45	Triploid, cage	unpublished
West River, MD		457	247	Diploid, spat-on-shell bottom	
Wicomico River, MD		365	247	Diploid, spat-on-shell bottom	
Huangdun Bay, China	*Ostrea plicatula* (Chinese oyster)	265	100	Diploid, rope and intertidal bottom	Ferreira et al. 2008
Sanggou Bay, China	*Crassostrea gigas*	51	20	Diploid, rope	
Loch Creran, Scotland	(Pacific oyster)	94	50	Diploid, intertidal trestles	Ferreira et al. 2009
Valdivia Bay, Chile		286	100	Diploid, suspended longline	Silva et al. 2011
Tornagaleones Bay, Chile		346	100	Diploid, suspended longline	
Niebla Bay, Chile		245	100	Diploid, suspended longline	
Isla del Rey, Chile		260	100	Diploid, suspended longline	

## Footnotes

1 Uncertainty in the N load estimates is less than 15% based on the relative standard deviation of the last four load estimates made for GBP from 2003–2011 (PREP 2012; M. Daley Shattuck, UNH, pers. Comm.).

2 Diploid oysters can reproduce, triploid oysters are sometimes used for aquaculture because they cannot reproduce and thus grow faster.

3 This is currently being superseded in FARM by an Individual Based Model (IBM) approach (see https://longline.co.uk/aqua2019/), that is now being implemented in FARM. The trade-off is runtime, a typical FARM run which now takes 15–30 min will take around 13 h using the IBM approach.
