# Supplementary material for: Bioextractive Removal of Nitrogen by Oysters in Great Bay Piscataqua River Estuary, New Hampshire, USA
Source: Estuaries Coast. Author manuscript; Available in PMC 2021 Jan 1. (PMC6997951; doi:10.1007/s12237-019-00661-8)
Supplement: Supplement1 [file NIHMS1549222-supplement-Supplement1.docx]

# Supplemental material

# for

**Bioextractive removal of nitrogen by oysters in**

**Great Bay Piscataqua River Estuary, New Hampshire, USA**

Suzanne B. Bricker^1^*, Raymond E. Grizzle^2^, Philip Trowbridge^3^, Julie M. Rose^4^, Joao G. Ferreira^5^, Katharine Wellman^6^, Changbo Zhu^5^, Eve Galimany^4^, Gary H. Wikfors^4^, Camille Saurel^5^, Robin Landeck Miller^7^, James Wands^7^, Robert Rheault^8^, Jacob Steinberg^1^, Annie P. Jacob^1^, Erik D. Davenport^1^, Suzanne Ayvazian^9^, Marnita Chintala^9^, and Mark A. Tedesco^10^

^1*^ National Centers for Coastal Ocean Science, Silver Spring, MD, corresponding author [Suzanne.bricker@noaa.gov](mailto:Suzanne.bricker@noaa.gov) (email), +301-723-3020 x139 (ph), +301-713-4388 (fax); [annie.jacob@noaa.gov](mailto:annie.jacob@noaa.gov), [eric.davenport@noaa.gov](mailto:eric.davenport@noaa.gov), [steinberg.jake@gmail.com](mailto:steinberg.jake@gmail.com) (now at School of Oceanography, University of Washington, 7616 Latona Ave NE, Seattle, WA 98115 )

^2^ Department of Biological Sciences, Jackson Estuarine Laboratory, 85 Adams Point Road, Durham, N H; [ray.grizzle@unh.edu](mailto:ray.grizzle@unh.edu)

^3^ New Hampshire Dept. of Environmental Services, Durham, NH 03824, [philt@sfei.org](mailto:philt@sfei.org) (now at Connecticut Department of Energy and Environmental Protection, 79 Elm St, Hartford, CT 06106  [Philip.Trowbridge@ct.gov](mailto:Philip.Trowbridge@ct.gov))

^4^ NOAA Fisheries NEFSC Milford Laboratory, 212 Rogers Avenue, Milford, CT 06460; [Julie.Rose@noaa.gov](mailto:Julie.Rose@noaa.gov), [Gary.Wikfors@noaa.gov](mailto:Gary.Wikfors@noaa.gov), [galimany@icm.csic.es](mailto:galimany@icm.csic.es)

^5^ Environmental Engineering, Faculty of Sciences and Technology, Universidade Nova de Lisboa, Portugal; [joao@hoomi.com](mailto:joao@hoomi.com), [changbo@scsfri.ac.cn](mailto:changbo@scsfri.ac.cn), [c](mailto:camillesaurel@hotmail.com)sau@aqua.dtu.dk

^6^ Northern Economics, Inc., 1455 NW Leary Way, Suite 400, Seattle WA 98107, [Katharine.Wellman@norecon.com](mailto:Katharine.Wellman@norecon.com)

^7^ HDR | HydroQual, 1200 MacArthur Boulevard, Mahwah, NJ 07430; [Robin.Miller@hdrinc.com](mailto:Robin.Miller@hdrinc.com), [James.Wands@hdrinc.com](mailto:James.Wands@hdrinc.com)

^8^ 1121 Mooresfield Rd., Wakefield, RI 02879; East Coast Shellfish Growers Association, [bob@ecsga.org](mailto:bob@ecsga.org)

^9^ EPA, ORD, National Health and Environmental Effects Research Laboratory, Atlantic Ecology Division, 27 Tarzwell Dr., Narragansett, RI 02882, [Ayvazian.Suzanne@epa.gov](mailto:Ayvazian.Suzanne@epa.gov) , [Chintala.Marty@epa.gov](mailto:Chintala.Marty@epa.gov)

^10^ EPA Long Island Sound Office, Government Center, Suite 9-11, 888 Washington Blvd., Stamford, CT, 06904-2152, [tedesco.mark@epa.gov](mailto:tedesco.mark@epa.gov)

Number of pages: 13

Number of tables: 6

Number of figures: 4

### Estimated costs for oyster aquaculture production

A study of the impact of 4 types of financing (i.e. self-finance, traditional bank loan, specialized loan for aquaculture that features an interest-only period and partial-principle forgiveness, and payment for nutrient removal ecosystem services) on the profitability success of aquaculture operations in Chesapeake Bay included itemization of costs incurred by oyster farm operations (Table 1). The study included analysis of bottom and water column (cage) culture operations. Although separate models were developed for oyster bottom-culture and water-column culture methods, some data and model assumptions are common to both types of operations (Table 1). The costs to Chesapeake Bay oyster growers are assumed to be relevant to the GBP oyster growers though costs for specific items may vary dependent on type (bottom vs water column) and size of operation; this is meant to provide information about other costs that could be incurred by GBP oyster growing operations.

Table 1. Common values used in model analysis of profitability calculations for bottom-culture and water-column oyster production in the Maryland Chesapeake Bay. (from Parker 2019)

| **Operating Cost Assumptions** | **Value** |
| --- | --- |
| Market-Size Oysters Per Bushel | 275 |
| Retail Containers for Half-Shell Market | 100 count box |
| Cost Of Retail Containers for Half-Shell Market | $1.00 per box |
| General Labor Rate | $12.50 per hour |
| Supervisory/Owner Labor Rate | $20.00 per hour |
| Supervisory/Owner Operator Labor Hours Per Week | 40 |
| Unemployment Insurance Tax | 2.6% of payroll |
| Federal Insurance Contributions Act (FICA) | 6.2% of payroll |
| Workman's Comp | 5% of payroll |
| General Liability Insurance | $1,000 per $150,000 in revenue per year |
| Boat Insurance | $600 per boat per year |
| Auto Insurance | $683 per auto per year |
| Repairs and Maintenance | 1% of variable costs – employment expenses |
| Overhead | 3% of variable costs |

### Costs of reef restoration in Great Bay Piscataqua River Estuary

Restoration of oyster populations has been a major goal for New Hampshire management agencies since the 1990s (NHEP 2000). The most widely used technique for restoration of oyster reefs in most areas has been the placement of shell or other "cultch" material directly onto existing reefs (or areas thought to be potentially productive oyster bottom) to provide suitable substrate for natural settlement of spat. This well-tested method has been shown to be effective as well as relatively inexpensive (Table 2). Costs for reef restoration in GBP are higher than costs in Chesapeake Bay where restoration costs are $25,000 per acre for comparable spat-on-shell method of restoration. However, in locations where bottom conditions require addition of substrate the cost increases to $125,000 per acre (Maryland Interagency Oyster Restoration Workgroup of the Sustainable Fisheries Goal Implementation Team 2015).

Table 2: Estimated costs for restoration of 1 acre of oyster bottom based on budget for the present project. (from Grizzle et al. 2006)


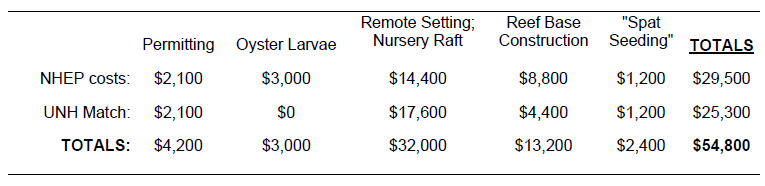


### The FARM model and Individual oyster model for *Crassostrea virginica*

This is a summary of the FARM model generally, and the calibration for Long Island Sound specifically; the GBP oyster modeling used the FARM model that was calibrated for Long Island Sound. Additional details can be found in Ferreira et al. (2007) and Bricker et al. (2015, 2018).

The Farm Aquaculture Resource Management (FARM) model (Figure 1) combines physical and biogeochemical models, bivalve growth models, and screening models to determine shellfish production and for eutrophication assessment at the farm scale (Ferreira et al. 2007). Water properties are transported both horizontally and vertically in the model, but the vertical component only applies to suspended culture. The model is driven by peak (i.e. mid-tide) current speeds measured *in situ* for both spring and neap tides, and uses an interpolation to generate the full semi-diurnal (or diurnal, where applicable) cycle for both height and velocity, and the change of amplitude through the lunar cycle. Velocities are not residuals, and the tidal height and velocity are calculated explicitly for each model timestep (Ferreira et al., 2007). The model calculates the phytoplankton and detrital carbon removed by shellfish (gross) as water and ‘food’ passes through the lease area (Figure 2), then converts those values to nitrogen and deducts losses due to pseudofeces, feces, excretion, mortality, and spawning. The mass balance provides a value for net removal of nitrogen from the water column by the population of oysters, which effectively equates to a drawdown of phytoplankton, i.e. of one of the primary symptoms of eutrophication (Figure 4). The general formulation used in FARM for modelling pelagic state variables in a suspended culture system is given in Eq. (1):


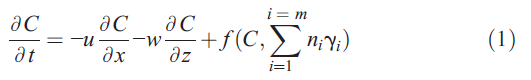


Where:


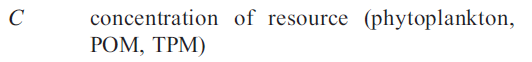

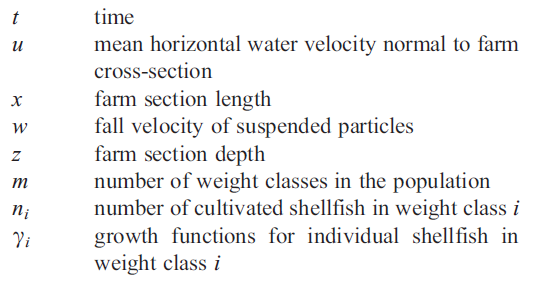


The third term in Eq. (1) is a general representation of sinks and sources associated with shellfish growth. This term may be a sink for e.g. DO or CHL, a source e.g. for excreted NH_4_, or both for e.g. POM and other particulate matter, which may be removed during ingestion and returned to the system as pseudofaeces and faeces.


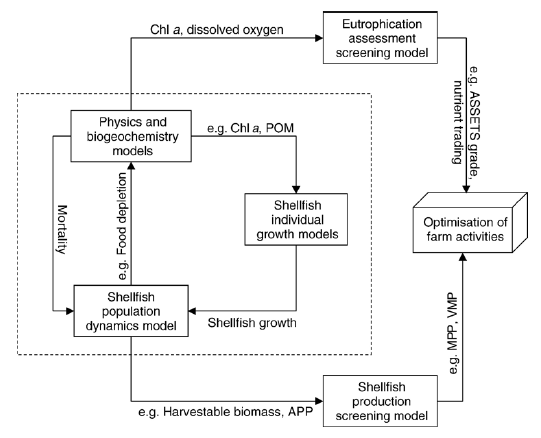


Figure 1. Conceptual scheme of the various components of the FARM model. The model core is within the dotted rectangle, the two screening models are external. (from Ferreira et al. 2007)


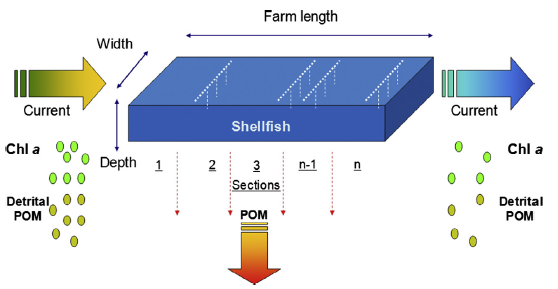


Figure 2: Farm layout (bottom culture) for the Farm Aquaculture Resource Management Model. Chl a = chlorophyll a, POM = particulate organic matter. (from Ferreira et al. 2007)

The model relies on an individual model for oyster growth and oyster associated environmental effects in order to simulate oyster aquaculture and bioextraction. The model is calibrated to a waterbody using data on individual local oyster growth rates. An individual model for the Eastern oyster (diploid) was developed using the AquaShell generic framework (Silva et al., 2011) which uses a net energy balance approach with morphometric formulations adjusted to the Long Island Sound study site to simulate local oyster growth (Bricker et al., 2015, 2018). The individual oyster growth model for *Crassostrea virginica* was calibrated and validated using experimental and field results from Bricker et al. (2015), together with data from Loosanoff (1947), Loosanoff and Nomejko (1949), Bricelj et al. (1992), Shaw and McCann (1963), and Zarnoch and Schreibman (2012). Experiments showed that oysters cleared 3.27-4.26 L hr^-1^ g dry weight^-1^, and assimilation efficiencies of 53-55% (Galimany et al., 2011). There was a good fit between the model predicted shell length (mm), total fresh weight (g) and tissue dry weight (g), observed data from Bricker et al. (2015) and the literature. The calibrated and validated individual model was integrated into the FARM model to simulate production and changes in relevant environmental parameters (e.g. DO, CHL, NH_4_) associated with the oyster farm (Figure 3). FARM incorporates all required processes and the models build those into a population model that includes losses from spawning and mortality.


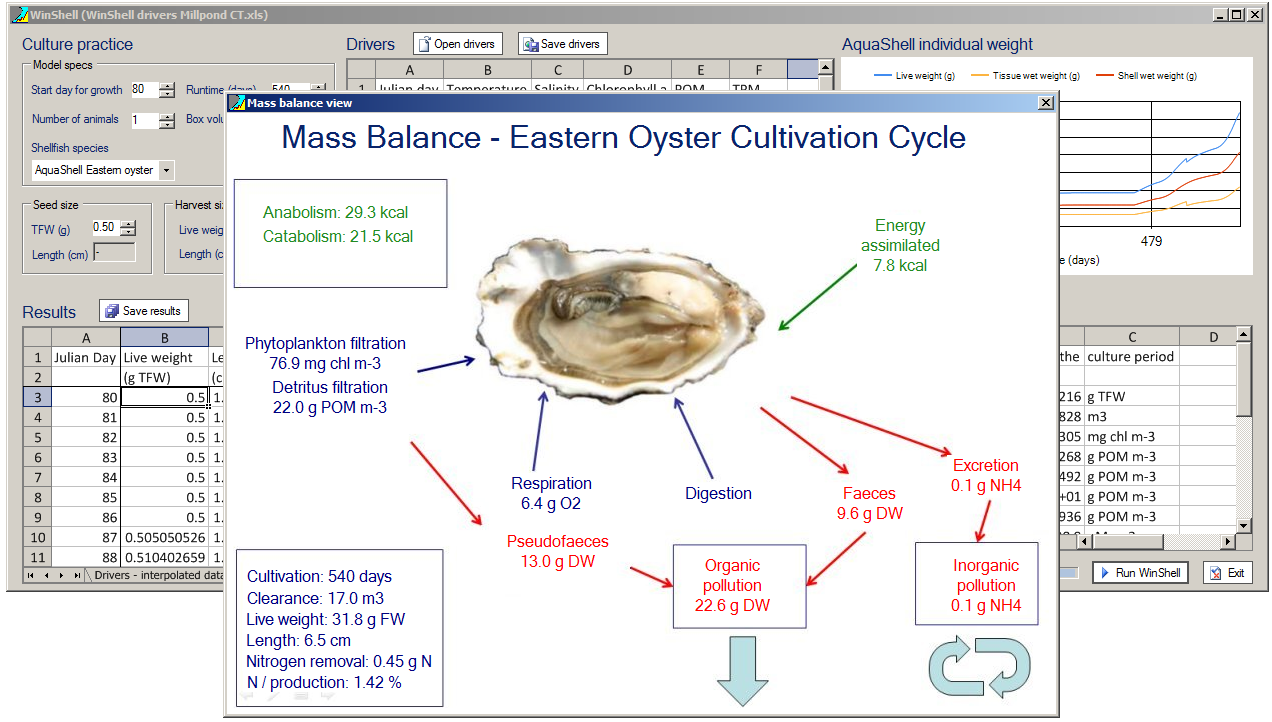


Figure 3: AquaShell Eastern oyster growth model example - Growth simulation mass balance. Oysters feed on algae / detritus, excrete and produce feces and pseudofeces. This mass balance of filtration uptake and losses from excretion, feces, pseudofeces, mortality and spawning provides production and environmental effects allowing population scale models to examine top-down control of eutrophication. (From Bricker et al. 2015)


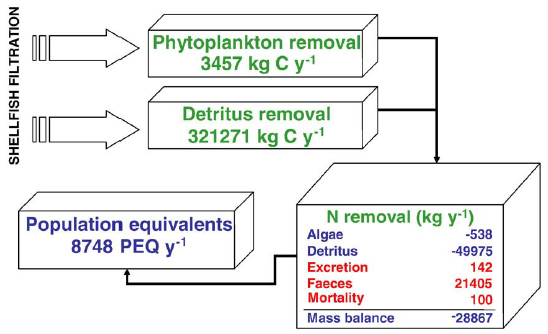


Figure 4: Mass balance and nutrient emissions for potential nutrient credit trading for clam aquaculture in Ria Formosa. Note that N removal is based on the balance of ingestion of phytoplankton and detritus minus excretion, feces, and mortality. (from Ferreira et al. 2009)

### Comparative costs for various categories of nitrogen removal strategies

A previous study (Rose et al. 2015) compared information on cost-effectiveness of available best management practices from eight studies and converted to a common currency (i.e., USD lb^-1^ nitrogen removed). Nitrogen removal strategies were grouped into one of six categories: shellfish, agricultural, urban stormwater, wastewater treatment upgrades, wetlands, and other (Table 3). The range of reported cost-effectiveness was several orders of magnitude for each category of best management practices. The range of potential costs for shellfish aquaculture as a nitrogen removal strategy was similar to that of the other best management practices.

Table 3: A summary of reported costs for six categories of nitrogen removal strategies. Reported costs have all been converted to USD lb^-1^ nitrogen. (from Rose et al. 2015).


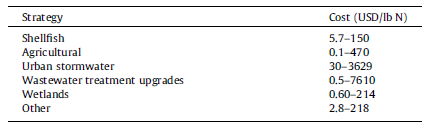


### Comparative removal efficiencies for various categories of nitrogen removal strategies

A previous study compared nitrogen removal rates from several best management practice strategies where data were available (Stephenson et al. 2010).

Removal efficiencies varied for agricultural BMPs depending on location within the part of the Chesapeake Bay watershed that is within the state of Virginia. Approved rates were determined for each of five watershed ‘‘basins’’, and within each basin on the western shore of Virginia, for BMPs that were located to the east or to the west of Interstate 95. Minimum and maximum annual nitrogen removal (in terms of lbs acre^-1^) for the five approved agricultural BMPs show a range of expected nitrogen removal rates (Table 4a). Nitrogen removal rates by shellfish farms compare very favorably, on a per-acre basis, to expected nitrogen removal by agricultural best management practices.

Table 4a: Nitrogen removal by agricultural best management practices in the Chesapeake Bay watershed, as approved by the Virginia Department of Environmental Quality (from Stephenson et al. 2010).


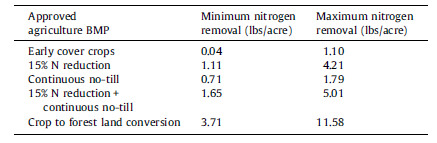


The University of New Hampshire Stormwater Center has implemented a variety of types of stormwater control measures (SCMs) in a controlled setting on their campus allowing scientists to quantify performance and cost of commonly-used SCMs and compare among the traditional and low impact design categories. We combined this information to calculate annual nitrogen removal in terms of lbs acre^-1^ for comparison to the agricultural BMPs and shellfish farms (Table 4b). The stormwater control measures that had no detectable nitrogen removal (vegetated swales, sand filters, and porous asphalt targeted total suspended solids and/or phosphorus rather than nitrogen). In general, nitrogen removal rates by stormwater control measures were higher, on a per-acre basis, than those reported for agricultural best management practices (Table 4a). Stormwater control measures that demonstrated nitrogen removal were in the same approximate range as those observed for shellfish farms which compared favorably to removal by stormwater control measures.

Table 4b: Nitrogen removal by different types of stormwater control measures, installed at the University of New Hampshire Stormwater Center (data from Houle et al. 2013 as summarized by Rose et al. 2015).


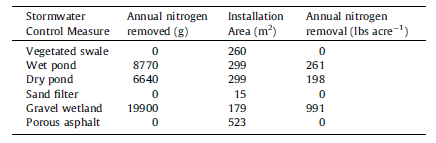


### Calculation of Point Source Reductions and Value of removal

The method of Evans (2008) was used to determine the WWTP costs for use a proxy value for the nitrogen (N) removed by oyster bioextraction (aquaculture and restored reefs) in Great Bay Piscataqua River Estuary. This excerpt from Evans (2008) describes the method of determination of N removal values for a variety of WWTPs of varying levels of treatment. While this describes the method for Long Island Sound WWTPs, the same approach was used for GBP using local WWTP costs from Kessler (2010) and costs were updated to 2013 dollars to adjust for inflation using the Engineering News Record Construction Cost Index. For more detail the reader is directed to Evans (2008) and Kessler (2010).

Excerpt adapted from Evans (2008):

Data were compiled on current wastewater characteristics (i.e., discharge flows and

concentrations) for 142 treatment plants located throughout the basin. For the purposes of this particular analysis, estimates were made of the potential nitrogen reductions that could be achieved by bringing all treatment plants outside of Connecticut to three specific ‘levels’ or target concentrations (i.e. 8 mg/l, 5 mg/l, 3 mg/l). For the first level (Level 1), an estimate was made of the reduction that could be achieved by bringing all plants with current discharge concentrations above 8 mg/l to a concentration of 8 mg/l. For the next two levels, similar calculations were made of potential reductions that would be obtained by upgrading plants from 8 to 5 mg/l, and then from 5 to 3 mg/l. The estimated basin-wide load reductions based on this approach are shown in Table 5.

Table 5: Estimated point source load reductions (from Evans 2008).


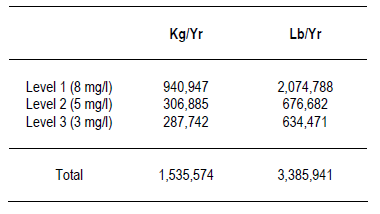


As given previously, the current estimated point source load delivered to Long Island Sound is 4,601,149 kg/yr (or 10,145,543 lb/yr). Given the values in Table 5, the current point source load from the entire basin could be reduced by about 33.4% (1,535,574 / 4,601,149) if all treatment plants above Connecticut were brought to a discharge concentration of 3 mg/l of total nitrogen. If only the point sources outside of Connecticut are considered (which contribute an annual load of about 2,054,916 kg), then the maximum potential load reduction would be 74.7% (1,535,574 / 2,054,916).

The costs associated with upgrading treatment plants for each of the treatment levels described above was accomplished using an approach previously developed as part of a study to estimate the cost of plant upgrades in the Chesapeake Bay Basin (Chesapeake Bay Program, 2002). In the latter study, various regression equations were developed to estimate total capital costs and early overhead and maintenance (O&&M) costs associated with treatment plants of different sizes (i.e., design discharge flows). More specifically, different equations were developed for each of the same three treatment levels used in this current study (i.e., existing effluent concentration to 8 mg/l, 8 mg/l to 5 mg/l, and 5 mg/l to 3 mg/l). Additional descriptions of the Chesapeake Bay study regressions also applied in this study are provided below.

*Note that the regression equations described below are based on costs in 2000 and only consider the cost of upgrading from one level to the next, and do not include the costs incurred for achieving the previous treatment level.*

For all treatment plants independent of size, the following equation was used to estimate total capital costs associated with Level 1 plant upgrades:

*Cost (in dollars)* = 2023829 + 704350.8039*Q* − 5986.733*Q*^2^

where *Q* = design flow rate (million gallons per day (mgd))

In the Chesapeake Bay study (as well as this one), annual O&M costs were assumed to be equal to 2% of the estimated total capital cost for each plant.

In the case of Level 2 upgrades, two different sets of cost equations were used depending on the design flow (in mgd) for each plant. The corresponding total capital cost and O&M equations for plants with design flows of 1 mgd or less are as follows:

*Capital Cost (in thousands of dollars)* = 967.06Q + 144.4

*Annual O&M Costs (in dollars)* = 24636Q + 4582.1

For plants with design flows greater than 1 mgd, the following equations were used:

*Capital Cost (in thousands of dollars)* = 386.01Q + 864.83

*Annual O&M Costs (in dollars)* = 13383Q + 19021

For Level 3 upgrades, the equations used were:

For plants with design flows of 1 mgd or less:

*Capital Cost (in thousands of dollars)* = 1061.7Q + 205.83

*Annual O&M Costs (in dollars)* = 24636Q + 4582.1

For plants with design flows greater than 1 mgd, the equations the following equations were used:

*Capital Cost (in thousands of d* = 386.01Q + 864.83

*Annual O&M Costs (in dollars)* = 13383Q + 19021

Since the Chesapeake Bay study used as a basis for cost estimation was completed using construction costs computed for the current study were increased by a factor of 18.1% to represent estimated construction cost increases from 2000 to 2007. (This estimated cost factor was obtained from information available on the Engineering News Record web site

(http://enr.construction.com/features/coneco/recentindexes.asp)). The updated total capital costs and annual O&M costs associated with each level of treatment are summarized in Table 6. Those wishing to know more about the specific data and methodologies used to develop the initial regression equations are referred to the original Chesapeake Bay study report (Chesapeake Bay Program, 2002).

Table 6: Estimated costs for upgrading all treatment plants outside of Connecticut. Note that although Evans (2008) updated to reflect 2007 dollars, our GBP study updated to reflect 2013 dollars.


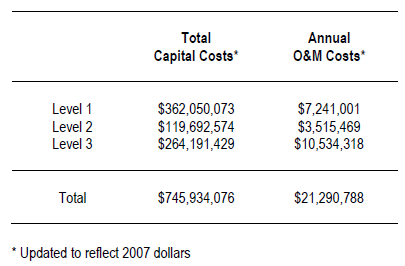


## **References**

Bricelj, V.M., S.E. Ford, F.J. Borrero, F.O. Perkins, G. Rivara, R.E. Hillman, R.A. Elston, and J. Chang 1992. Unexplained mortalities of hatchery-reared, juvenile oysters, *Crassostrea virginica* (Gmelin). *J Shellfish Res.* 11: 331–347

Bricker, S.B., J.G. Ferreira, and T. Simas. 2003. An Integrated Methodology for Assessment of Estuarine Trophic Status. *Ecol. Modelling* 169: 39-60.

Bricker SB, Longstaff B, Dennison W, Jones A, Boicourt K, Wicks C, Woerner J. 2007. Effects of Nutrient Enrichment in the Nation’s Estuaries: A Decade of Change, National Estuarine Eutrophication Assessment Update. NOAA Coastal Ocean Program Decision Analysis Series No. 26. National Centers for Coastal Ocean Science, Silver Spring, MD. 322 pp.

Bricker, S.B., J.G. Ferreira, C. Zhu, J.M. Rose, E. Galimany, G.H. Wikfors, C. Saurel, R. Landeck Miller, J. Wands, P. Trowbridge, R.E. Grizzle, K. Wellman, R. Rheault, J. Steinberg, A. P. Jacob, E. D. Davenport, S.Ayvazian, and M. A. Tedesco. 2015. *An* [*Ecosystem Services Assessment using bioextraction technologies for removal of nitrogen and other parameters in Long*](https://www.coastalscience.noaa.gov/publications/detail?resource=O0hdKo2k2hSWOxLGFRcu/feoeR4U4RM469gysLLrINQ=) *Island Sound and Great Bay/Piscataqua Region*. NCCOS Coastal Ocean Program Decision Analysis Series No. 194. NOAA NCCOS, Silver Spring, MD and US EPA ORD, Atlantic Ecology Division, Narragansett, RI. 220 pp + 3 appendices

Bricker, S.B., J.G. Ferreira, C. Zhu, J.M. Rose, E. Galimany, G.H. Wikfors, C. Saurel, R.L. Miller, J. Wands, P. Trowbridge, R.E. Grizzle, K. Wellman, R. Rheault, J. Steinberg, A.P. Jacob, E.D. Davenport, S. Ayvazian, M. Chintala, and M.A. Tedesco. 2018. The role of shellfish aquaculture in reduction of eutrophication in an urban estuary. *Environmental Science & Technology* 52: 173-183.

Chesapeake Bay Program. 2002. Nutrient Reduction Technology Cost Estimations for Point Sources in the Chesapeake Bay Watershed. 132 pp.

Evans, B.M. 2008. An Evaluation of Potential Nitrogen Load Reductions to Long Island Sound from the Connecticut River Basin. Report Submitted to New England Interstate Water Pollution Control Commission. University Park, PA: Penn State Institutes of Energy and the Environment.

Ferreira, J.G., J.S. Hawkins, and S.B. Bricker. 2007. Farm-scale assessment of shellfish aquaculture in coastal systems – The Farm Aquaculture Resource Management (FARM) model. *Aquaculture* 264: 160-174.

Ferreira, J.G., A. Sequeira, A.J.S. Hawkins, A. Newton, T.D. Nickell, R. Pastres, J. Forte, A. Bodoy, S.B. Bricker. 2009. Analysis of coastal and offshore aquaculture: Application of the FARM model to multiple systems and shellfish species. *Aquaculture* 292: 129–138.

Galimany, E., M. Ramón, and I. Ibarrola. 2011. Feeding behavior of the mussel *Mytilus galloprovincialis* (L.) in a Mediterranean estuary: a field study. Aquaculture 314: 236–243.

Grizzle, R.E, J. Greene, H. Abeels, and M. Capone. 2006. Reef structure alternatives for restoration of oyster (*Crassostrea virginica*) populations in New Hampshire. A Final Report to The New Hampshire Estuaries Project.

Houle, J., Roseen, R., Ballestero, T., Puls, T., Sherrard, J., 2013. A comparison of maintenance cost, labor demands, and system performance for LID and conventional stormwater management. *J. Environ. Eng*. 139, 932–938.

Kessler, K. 2010. Analysis of Nitrogen Loading Reductions for Wastewater Treatment Facilities and Non-Point Sources in the Great Bay Estuary Watershed. Appendix E. Concord, NH: New Hampshire Department of Environmental Services.

Loosanoff, V.L., and C.A. Nomejko. 1949. Growth of oysters, *C. virginica*, during different months. *Biol. Bull*. (Woods Hole) 97(1):82-94.

Loosanoff, V.L. 1947. Growth of oysters of different ages in Milford Harbor, Connecticut. Southern Fisherman, January 1947: 222-225.

Maryland Interagency Oyster Restoration Workgroup of the Sustainable Fisheries Goal Implementation Team. 2015. [Tred Avon River Oyster Restoration Tributary Plan](https://chesapeakebay.noaa.gov/images/stories/habitats/april2015tredavontribplan.pdf): A blueprint for sanctuary restoration.

NHEP. 2000. New Hampshire Estuaries Project Management Plan. Office of State Planning, Portsmouth, NH.

Parker, M.D. 2019. Effects of different capital sources on Maryland oyster aquaculture operations. Dissertation submitted to the Faculty of the Graduate School of the University of Maryland, College Park, in partial fulfillment of the requirements for the degree of Doctor of Philosophy in Marine Estuarine Environmental Sciences.

Rose, J.M., S.B. Bricker, J.G. Ferreira. 2015. Modeling shellfish farms to predict harvest-based nitrogen removal. *Marine Pollution Bulletin* 453: 135-146.

Silva, C., J.G. Ferreira, S.B. Bricker, T.A. DelValls, M.L. Martín-Díaz, E. Yáñez. 2011. Site selection for shellfish aquaculture by means of GIS and farm-scale models, with an emphasis on data-poor environments. *Aquaculture* 318: 444–457.

Shaw, W. and J.A. McCann. 1963. Comparison of growth of four strains of oysters raised in Taylors Pond, Chatham, Mass. U.S. Fish and Wildlife Service, Fishery Bulletin 53(1): 11-17.

Stephenson, K., Aultman, S., Metcalfe, T., Miller, A., 2010. An evaluation of nutrient nonpoint offset trading in Virginia: a role for agricultural nonpoint sources? *Water Resour. Res*. 46, W04519.

Zarnoch, C.B. and M.P. Schreibman. 2012. Growth and Reproduction of Eastern Oysters, *Crassostrea Virginica*, in a New York City Estuary: Implications for Restoration. Urban Habitats vol. 7. <http://www.urbanhabitats.org/v07n01/easternoysters_full.html>.
